# Dual life history of *Biscogniauxia barriae* sp. nov. (*Xylariales, Graphostromataceae*) as a Douglas-fir foliar endophyte and hardwood saprotroph

**DOI:** 10.3897/mycokeys.140.206138

**Published:** 2026-09-08

**Authors:** Joey B. Tanney, Nicolas Feau, Yu-Ming Ju

**Affiliations:** 1 Pacific Forestry Centre, Canadian Forest Service, Natural Resources Canada, Victoria, BC, Canada Pacific Forestry Centre, Canadian Forest Service, Natural Resources Canada Victoria Canada; 2 Institute of Plant and Microbial Biology, Academia Sinica, Nankang, Taipei 115, Taiwan Institute of Plant and Microbial Biology, Academia Sinica Taipei Taiwan

**Keywords:** Foliar mycobiome, fungal genomics, *
Graphostromataceae
*, one new species, phylogeny, secondary metabolites, *
Xylariales
*

## Abstract

*Biscogniauxia* species are typically recognized by carbonaceous stromata on woody substrates, but some also occur as asymptomatic endophytes and may shift ecological roles across hosts and substrates. Here, *Biscogniauxia barriae***sp. nov**. is described, first isolated as a foliar endophyte from asymptomatic Douglas-fir (*Pseudotsuga menziesii*) needles in coastal British Columbia and later found producing stromata on dead hardwoods, including bigleaf maple (*Acer macrophyllum*), red alder (*Alnus rubra*), and hawthorn (*Crataegus* sp.). Phylogenetic analyses of ITS, *BenA*, and *RPB2* resolved *B. barriae* as a distinct, well-supported lineage within *Biscogniauxia* s.s., sister to *B. capnodes* and allied with a clade including *B. anceps* and *B. nummularia*. The species is characterized by applanate to pulvinate, carbonaceous stromata with coarsely papillate ostioles and dark brown, broadly ellipsoid ascospores with a conspicuous straight germ slit; a nodulisporium-like asexual morph is produced in culture. The highly complete 42.7 Mb draft genome contained 10,986 predicted protein-coding genes and was broadly comparable to available *Biscogniauxia* genomes. Orthogroup analyses indicated extensive conservation of protein-coding gene content, while identification of a MAT1-2 idiomorph suggests heterothallism. The discovery of *B. barriae* through both Douglas-fir endophyte surveys and hardwood stromatal collections highlights a dual life history linking conifer foliar endophytism with hardwood-associated saprotrophy or possible latent pathogenicity. Strong inhibition of the *in vitro* growth of *Nothophaeocryptopus gaeumannii*, the causal agent of Swiss needle cast, by culture-filtrate extracts of *B. barriae* suggests potential ecological relevance within the Douglas-fir needle mycobiome.

## Introduction

The genus *Biscogniauxia* (*Graphostromataceae*, *Xylariales*) includes more than 60 species described worldwide from woody hosts across divergent orders, including *Fagales*, *Lamiales*, *Laurales*, *Sapindales*, and *Vitales* (USDA Fungal Databases 2025). Although typically encountered through their conspicuous carbonaceous stromata on woody substrates, some *Biscogniauxia* species also occur as asymptomatic endophytes and may shift ecological roles depending on host condition and environmental stress. Several species are recognized as latent pathogens, establishing endophytic infections that become pathogenic when their hosts are subjected to drought and high-temperature stress (Nugent et al. 2005; Vannini et al. 2009). Associated diseases include beech charcoal canker (*B. nummularia*), beech bark tarcrust (*B. destructiva*), oak charcoal disease (*B. mediterranea*), and charcoal canker of pear, plum, and quince (*B. rosacearum*). These diseases are named for the conspicuous, dark, crust-like stromata that emerge from the bark of infected trees.

Morphologically, *Biscogniauxia* species are characterized by widely effuse to raised-discoid, carbonaceous stromata with distinct ostioles along the surface and perithecia that are usually embedded in a single layer (Ju and Rogers 2001). The eight-spored asci typically possess amyloid apical rings and contain ellipsoid, brown ascospores with or without straight or sigmoidal germ slits. Anamorphs are nodulisporium-like, with cylindrical, finely roughened conidiogenous cells that bear poroid conidial secession scars across the apical region. Conidia are hyaline, smooth, ellipsoid to clavate or cylindrical, dry, and produced holoblastically in a sympodial sequence.

Like many other members of *Xylariales*, some species of *Biscogniauxia* exhibit broad endophytic host ranges while forming stromata on a narrower subset of hosts or tissues and under certain environmental or physiological conditions (Petrini and Petrini 1985; Ibrahim et al. 2020). For example, *B. nummularia* has been isolated from stromata on dead and living *Fagus sylvatica* trees as well as from nearby asymptomatic leaves of the sedge *Carex brevicollis* (Zabalgogeazcoa et al. 2015). The host impact of foliar colonization by *Biscogniauxia* remains poorly understood, although species of the genus have occasionally been associated with foliage or foliar disease symptoms, including *B. sinensis* from blighted *Pinus thunbergii* needles (Qiao et al. 2024) and *B. rosacearum* from diseased *Myrtus communis* leaves (Khadraoui et al. 2025).

Graham et al. (2025) recently reported a putatively novel species of *Biscogniauxia* isolated as an endophyte from asymptomatic needles of Douglas-fir (*Pseudotsuga menziesii*) on Vancouver Island, British Columbia, Canada. Two strains of this species significantly inhibited the *in vitro* growth of *Nothophaeocryptopus gaeumannii*, the causal agent of Swiss needle cast of Douglas-fir, suggesting potential ecological relevance within the Douglas-fir needle mycobiome. Here, the subsequent discovery of corresponding stromata on dead bigleaf maple (*Acer macrophyllum*), red alder (*Alnus rubra*), and hawthorn (*Crataegus* sp.) in coastal British Columbia is reported. Based on phylogenetic, morphological, and genomic evidence, this fungus is described as *B. barriae* sp. nov., and its dual life history as a Douglas-fir foliar endophyte and hardwood-associated saprotroph is discussed.

## Methods

### Field collecting, isolation, and morphological analyses

Axenic strains of *Biscogniauxia* sp. nov. were isolated as endophytes from surface-sterilized needles of Douglas-fir as described by Graham et al. (2025). Corresponding stromata were collected from dead *Acer macrophyllum* stems and branches, a dead standing *Alnus rubra* stem, and *Crataegus* sp. branches on southern Vancouver Island. Mono-ascospore strains were generated using a flame-sterilized scalpel to expose asci, which were then streaked on a Petri dish containing 2% malt extract agar (MEA; 20 g Bacto malt extract, Difco Laboratories, Sparks, Maryland, USA; 15 g agar, EMD Chemicals Inc., New Jersey, USA; 1 L distilled water). Using a sterile insect pin, individual ascospores were picked up and placed on MEA. Plates were incubated at 20 °C in the dark and monitored for germination.

Strains of *Biscogniauxia* sp. nov. are maintained in J.B. Tanney’s culture collection (JTCC) as mycelial plugs in sterile deionized water at 4 °C and in 20% (v/v) glycerol in sterile deionized water at −80 °C. Representative strains were deposited in the Canadian Collection of Fungal Cultures (CCFC, Agriculture and Agri-Food Canada, Ottawa, Canada). Specimens of *Biscogniauxia* sp. nov. were accessioned in the Forest Pathology Herbarium at the Canadian Forest Service’s Pacific Forestry Centre (DAVFP, Victoria, BC, Canada).

Micromorphological features of colonies and both asexual and sexual morphs were examined and measured from living material (except for specimen WSP72952) mounted in tap water. Measurements are reported as ranges calculated from the mean ± SD for each parameter, with minimum and maximum values given in parentheses. To assess diameter growth and colony morphology, two strains (DAOMC 256931 and DAOMC 256939) were each inoculated via 5-mm mycelial plugs onto 10-cm-diameter Petri dishes containing MEA, oatmeal agar (OA; extract of 30 g/L boiled oatmeal, 15 g agar, and 1 L distilled water), or potato dextrose agar (PDA; Difco Laboratories, Detroit, MI, USA). Plates were incubated at 20 °C upside down in the dark and measured after 7 d. Inoculations were conducted in triplicate for each medium. Observations were conducted using a Leica DM4 B light microscope (Leica Microsystems CMS GmbH, Wetzlar, Germany) and a Leica M165 C stereomicroscope equipped with a TL5000 Ergo light base (Leica Microsystems Ltd., Singapore). Images were captured with a Leica DFC450 C camera (light microscope) and a Leica DMC5400 camera (stereomicroscope), both operated using Leica LAS X v. 3.7.1 software. Photographic plates were assembled using Adobe Photoshop CC 2019 v. 23.0.1 (Adobe Systems, San Jose, California, USA).

### DNA extraction, PCR amplification, and sequencing

DNA was extracted from 2-week-old colonies on MEA using the DNeasy UltraClean Microbial Kit (Qiagen, Hilden, Germany) according to the manufacturer’s instructions. Four gene regions were amplified by PCR using the following primer pairs: the internal transcribed spacer rDNA region (ITS), V9G/LS266 (Masclaux et al. 1995; de Hoog and Gerrits van den Ende 1998); the nuclear 28S large ribosomal subunit (LSU), LR0R/LR5 (Vilgalys and Hester 1990; Rehner and Samuels 1995); a partial region of β-tubulin (*BenA*), Bt2a/Bt2b (Glass and Donaldson 1995); and a partial region of the RNA polymerase II second-largest subunit (*RPB2*), RPB2-5f2/RPB2-7cR (Liu et al. 1999; Sung et al. 2007). PCR reactions were performed according to Tanney et al. (2024).

### Phylogenetic analyses

Phylogenetic analyses were conducted using sequences of related *Graphostromataceae* taxa from newly obtained collections and GenBank (Table 1). To assess the placement of *Biscogniauxia* sp. nov. under the genealogical concordance phylogenetic species recognition concept, single-gene and concatenated alignments were generated for taxa represented by ITS, *BenA*, and *RPB2* sequences. The final dataset included 37 strains/specimens representing 28 taxa, with *Hypoxylon rubiginosum* (YMJ 24; *Hypoxylaceae*) and *Jackrogersella cohaerens* (YMJ 310; *Hypoxylaceae*) selected as outgroups. To increase taxon sampling, a supplementary expanded phylogenetic analysis was conducted using a concatenated ITS, *BenA*, and *RPB2* alignment of 93 strains/specimens, including 91 *Graphostromataceae* and two *Hypoxylaceae* outgroups. This dataset included taxa represented by one, two, or all three loci, with unavailable gene regions treated as missing data.

**Table 1.** Taxa, strain or specimen identifiers, GenBank accession numbers, and references for sequences used in phylogenetic analyses. A dash (—) indicates that a sequence was unavailable. Superscript ‘^T^’ indicates sequences derived from type or ex-type material.

**Taxon**	**Identifier**	**GenBank accession no**.			**Reference**
		**ITS**	***BenA***	***RPB2***	
*Biscogniauxia aff. uniapiculata*	GAB220	KY250378	—	—	Direct submission
*Biscogniauxia anceps*	CBS:149687	OQ990096	—	—	Crous et al. 2023
	K(M):201862	MZ159582	—	—	Direct submission
	YMJ 123 = BCRC34029	EF026132	AY951671	JX507777	Hsieh et al. 2005; Hsieh et al. 2010; Mirabolfathy et al. 2013
*Biscogniauxia arima*	YMJ 122^T^ = BCRC34030	EF026150	AY951672	GQ304736	Hsieh et al. 2005; Hsieh et al. 2010
*Biscogniauxia atropunctata*	G411	KM215648	—	—	Direct submission
	IUB:B.Whitaker:OTU41	MH178710	—	—	Direct submission
	KC2-031E5	KX589181	—	—	Direct submission
	NY8660c	HQ108028	—	—	Direct submission
	SGSGf17	EU715651	—	—	Sánchez-Ballesteros et al. 2000
	YMJ 128	JX507799	AY951673	JX507778	Hsieh et al. 2005; Mirabolfathy et al. 2013
*Biscogniauxia atropunctata var. intermedia*	B70M = GB 4796	AJ390412	—	—	Sánchez-Ballesteros et al. 2000
*Biscogniauxia barriae*	DAOMC:256931	PP734788	PQ871684	—	Graham et al. 2025
	DAOMC:256937	PP734786	—	—	Graham et al. 2025
	DAOMC:256939^T^	PZ618325	PZ617968	PZ617971	This study
	JTCC-1167	PP833123	PP840330	PP840332	Tanney et al. 2024
	JTCC-421	PZ618326	—	—	This study
	DAOMC:252811	PZ618327	PZ617969	PZ617972	This study
	JTCC-837	PZ618328	PZ617970	PZ617973	This study
	WSP72952	PZ618330	—	—	This study
*Biscogniauxia bartholomaei*	108898191	ON692782	—	—	Pinto 2001
	ATCC 38992	AF201719	—	—	Pinto 2001
	DAOMC:252803	PP833124	PP840331	PP840333	Tanney et al. 2024
	DAOMC:252804/JTCC-1542	PZ545728	PZ617974	—	This study
	DAOMC:252805/JTCC-1543	PZ618323	—	—	This study
*Biscogniauxia capnodes*	FS39	MF770835	—	—	Direct submission
	GAB038	KY250379	—	—	Direct submission
	S74	OR237607	—	—	Direct submission
	YMJ 138 = BCRC34032	EF026131	AY951675	JX507779	Hsieh et al. 2005; Hsieh et al. 2010; Mirabolfathy et al. 2013
*Biscogniauxia capnodes var. capnodes*	11682 = NZFS:4537	MH410019	—	—	Direct submission
*Biscogniauxia cf. petrensis*	RLC_1445.1_iNat_7002890	OQ878456	—	—	Vandegrift et al. 2023
*Biscogniauxia cinereolilacina*	CBS:104.96	MH862567	—	—	Vu et al. 2019
*Biscogniauxia citriformis*	YMJ 129 = BCRC34034	JX507801	AY951678	JX507781	Hsieh et al. 2005; Mirabolfathy et al. 2013
	YMJ 88113012	JX507800	AY951677	JX507780	Hsieh et al. 2005; Mirabolfathy et al. 2013
*Biscogniauxia cylindrispora*	YMJ 89092701^T^ = BCRC33717	EF026133	AY951679	JX507782	Hsieh et al. 2005; Hsieh et al. 2010; Mirabolfathy et al. 2013
*Biscogniauxia dendrobii*	MFLUCC 17-2607^T^	NR_172733	MK975826	—	Ma et al. 2020
*Biscogniauxia dicranopteridis*	HKAS 124928^T^	OR001813	—	OR001855	Chen et al. 2024
*Biscogniauxia formosana*	G-jav1-ITS2_OTU-0-366_2	KY588559	—	—	Direct submission
	YMJ 89032201^T^ = BCRC33718	JX507802	AY951680	JX507783	Ju and Rogers 2001; Mirabolfathy et al. 2013
*Biscogniauxia glaucae*	GMBC0007^T^	MT624046	MT622654	MT622652	Li et al. 2021
*Biscogniauxia granmo*	YMJ 135 = BCRC34035	JX507803	AY951681	JX507784	Hsieh et al. 2005; Mirabolfathy et al. 2013
*Biscogniauxia latirima*	YMJ 89101101^T^ = BCRC33729	JX507804	AY951682	JX507785	Hsieh et al. 2005; Mirabolfathy et al. 2013
	YMJ 90080703 = BCRC34036	EF026135	AY951683	JX507786	Hsieh et al. 2005; Mirabolfathy et al. 2013
*Biscogniauxia magna*	MFLU 18-0850	MW240616	—	—	Samarakoon et al. 2022
*Biscogniauxia mangiferae*	MFLU 18-0827^T^	MN337232	MN509782	PQ480023	Maharachchikumbura et al. 2016; Gunarathne et al. 2025
*Biscogniauxia marginata*	CBS 124505	KU684016	KU684124	KU684310	Li et al. 2021
	ZT-Myc-64233	MW489534	—	—	Direct submission
	KACC:410582	OR886237	—	—	Direct submission
	KoLRI_EL004708	MN844502	—	—	Direct submission
	KoRLI046050	MN341558	—	—	Direct submission
	LE 262845	JQ247198	—	—	Direct submission
*Biscogniauxia mediterranea*	Bx70	KT253502	KT253536	—	Raimondo et al. 2016
	Bx85	KT253503	KT253537	—	Raimondo et al. 2016
	F8012	AB693903	—	—	Direct submission
	Kern805	OP038087	—	OP095258	Travadon et al. 2022
	VH04	OR778793	—	—	Direct submission
	YMJ 147 = BCRC34037	EF026134	AY951684	GQ844765	Hsieh et al. 2010; Raimondo et al. 2016
*Biscogniauxia nothofagi*	NZFS 3015	MN007010	—	—	Direct submission
*Biscogniauxia nummularia*	F8422	ON332160	—	—	Direct submission
	H86 = CCF 3919	GQ428318	GQ428324	FR715504	Pažoutová et al. 2010; Raimondo et al. 2016
	MUCL 51395^T^	NR_153649	KX271241	KY624236	Wendt et al. 2018
*Biscogniauxia papillata*	BCC 36828^T^	NR_202565	PQ604605	—	Wongkanoun et al. 2026
*Biscogniauxia persica*	YMJ 1461^T^	JX507807	JX507796	JX507792	Mirabolfathy et al. 2013
*Biscogniauxia petrensis*	HKAS 102388	MW240615	—	—	Samarakoon et al. 2022
	ST253	LC685807	—	—	Direct submission
	X2P3HRa	ON921656	—	—	Direct submission
*Biscogniauxia pezizoides*	WSP73521	PQ227784	—	—	Brenken and Riebesehl 2025
	WSP73553	PQ227785	—	—	Brenken and Riebesehl 2025
*Biscogniauxia philippinensis var. microspora*	YMJ 89041101^T^ = BCRC33720	EF026136	AY951685	JX507787	Mirabolfathy et al. 2013; Raimondo et al. 2016
*Biscogniauxia repanda*	ATCC:62606	KY610383	—	—	Wendt et al. 2018
	B75A	AJ390418	—	—	Bashiri et al. 2022
*Biscogniauxia rosacearum*	Bx26 = CBS141046^T^	KT253493	—	—	Raimondo et al. 2016
	IRAN 4288C	MZ359664	—	—	Bashiri et al. 2022
*Biscogniauxia simplicior*	YMJ 136 = BCRC34038	EF026130	AY951686	JX507788	Mirabolfathy et al. 2013; Raimondo et al. 2016
*Biscogniauxia sinensis*	SC1 = CFCC59648	OR803753	OR832177	OR832187	Qiao et al. 2024
	SC3 = CFCC59650^T^	OR803755	OR832179	OR832189	Qiao et al. 2024
*Biscogniauxia uniapiculata*	YMJ 90080608 = BCRC34039	JX507805	AY951687	JX507789	Mirabolfathy et al. 2013; Raimondo et al. 2016
*Biscogniauxia whalleyi*	SWUF 13-85^T^	MW403821	MZ466386	MZ466387	Direct submission
*Biscogniauxia xishuangbannaensis*	KUMCC21-0672^T^	PP663077	—	PX521740	Direct submission
*Camillea broomeana*	GMBC0218	MW854657	—	MW855488	Direct submission
*Camillea obularia*	ATCC 28093	KY610384	KX271243	KY624238	Wendt et al. 2018
*Camillea tinctor*	CBS:203.56	OQ831988	—	KU684282	Direct submission
	MFLU 18-0786	MW240614	MW775575	MW342618	Samarakoon et al. 2022
	YMJ 363	JX507806	JX507795	JX507790	Mirabolfathy et al. 2013
*Cryptostroma corticale*	JTCC-1456	PP439175	PP474968	PP447284	Tanney et al. 2024
	JTCC-1458	PP439177	PP474969	PP447283	Tanney et al. 2024
*Graphostroma guizhouensis*	GMBC0219^T^	MW854659	—	MW855487	Direct submission
*Graphostroma platystomum*	CBS 270.87^T^	JX658535	HG934108	KY624296	Stadler et al. 2014; Koukol et al. 2015; Wendt et al. 2018
	CBS:146066	MT223799	MT223734	MT223680	Crous et al. 2020
*Hypoxylon rubiginosum*	YMJ 24 = BCRC34116	EF026143	AY951751	JX507791	Mirabolfathy et al. 2013; Raimondo et al. 2016
*Jackrogersella cohaerens*	YMJ 310 = BCRC34013	EF026140	AY951655	GQ844766	Hsieh et al. 2010
*Obolarina dryophila*	8409	GQ428316	GQ428321	—	Pažoutová et al. 2010
	H76	GQ428317	GQ428323	—	Pažoutová et al. 2010

Sequences were aligned using MAFFT (Katoh et al. 2019) and concatenated in Geneious Prime v. 2026.1.1 (Biomatters, Auckland, New Zealand). Phylogenetic trees were reconstructed with maximum likelihood (ML) using IQ-TREE v. 3.1.2 (Wong et al. 2026). Concatenated datasets were analyzed as partitioned alignments by locus, with the best-fit substitution model selected automatically for each partition based on Bayesian information criterion (BIC) scores calculated in ModelFinder (Kalyaanamoorthy et al. 2017). Node support was assessed with 1,000 ultrafast bootstrap (UFBoot) replicates and 1,000 SH-like approximate likelihood ratio test (SH-aLRT) replicates. Phylogenetic trees were visualized in Geneious Prime and edited with Adobe Illustrator v. 30.5.1 (Adobe Systems, San Jose, CA, USA). Supplementary data were deposited in Figshare under DOI https://doi.org/10.6084/m9.figshare.32841839.

### Whole genome sequencing and annotation

For DNA extractions, mycelial mats of *Biscogniauxia* sp. (DAOMC 256937) were produced by culturing the fungus in 125 mL of malt extract (ME) broth at 20 °C with shaking at 200 rpm for 4–5 weeks. Mycelium was then lyophilized for 48 h and ground in liquid nitrogen. For Illumina sequencing, genomic DNA was extracted using a cetyltrimethylammonium bromide–chloroform protocol with isopropanol precipitation (Zolan and Pukkila 1986). DNA concentration and purity were measured with a NanoDrop ND-1000 spectrophotometer (Thermo Scientific). Paired-end (150 bp) libraries were prepared with the NEBNext Ultra II DNA Library Prep Kit for Illumina (New England Biolabs) and sequenced at the Génome Québec Innovation Center (Montréal, Canada) on an Illumina HiSeq X platform. DNA extraction and library preparation for Oxford Nanopore (ON) sequencing were performed at the Plateforme d'Analyses Génomiques, Institut de Biologie Intégrative et des Systèmes, Université Laval (Quebec, Canada), as described in Feau et al. (2024). Briefly, high-molecular-weight DNA was isolated from ~30 mg of dried fungal mycelium using a chloroform–isopropanol extraction (Doyle and Doyle 1990). Approximately 3 μg of DNA was resuspended and enriched for large fragments using the Short Read Eliminator XS kit (SRE XS, PacBio). Multiplex libraries were constructed with 1 μg of DNA using the SQK-LSK109 and EXP-NBD104 kits (Oxford Nanopore Technologies). Sequencing was carried out on an R9.4.1 flow cell using a GridION instrument, and base calling was performed with Guppy v. 6.4.2 using the fast model. ON and paired-end Illumina reads were assembled into contigs with SPAdes v. 3.15 (Prjibelski et al. 2020) using default parameters. This draft genome assembly was polished with three rounds of Racon v. 1.4.3 (Vaser et al. 2017) using ON reads mapped with minimap2 (Li 2018), followed by seven iterations with Pilon v. 1.24 (Walker et al. 2014) using Illumina reads mapped with BWA (Li and Durbin 2009). Genome annotation was conducted with Funannotate v. 1.8 (Palmer and Stajich 2020) using protein evidence only, as no transcriptomic data were available. Protein sequences from *Biscogniauxia marginata*, *B. nummularia*, and *B. mediterranea* were provided as external evidence (Suppl. material 1). *Ab initio* gene prediction was performed with AUGUSTUS v. 3.5 (Stanke et al. 2008) using the pre-trained *Neurospora crassa* model, chosen for its phylogenetic proximity within *Sordariomycetes*. Completeness of the genome assembly and predicted gene set was evaluated with Compleasm (Huang and Li 2023) based on the fungi_odb10 Benchmarking Universal Single-Copy Orthologs (BUSCO) dataset (Manni et al. 2021). Comparative analysis of the protein-coding gene set of *B. barriae* sp. nov. with the protein-coding gene sets of other *Graphostromataceae* taxa was carried out using OrthoFinder v. 2.5.5 with default parameters (Emms and Kelly 2019).

### Divergence time estimation

Divergence time was estimated using BEAST v. 2.7.7 (Bouckaert et al. 2019) for *B. barriae* and five other members of *Graphostromataceae* (*B. nummularia*, *B. mediterranea*, *Biscogniauxia* sp. FL1348, *B. marginata*, and *Cryptostroma corticale*). *Biscogniauxia* sp. FL1348 is an undescribed endolichenic isolate recovered from a surface-sterilized thallus of *Cladonia evansii* collected at Archbold Biological Station, Florida, USA (Franco et al. 2022). To provide phylogenetic context and calibration points, the dataset was expanded to include five additional *Xylariales* (*Daldinia eschscholtzii*, *Hypoxylon rubiginosum*, *Eutypa lata*, *Entoleuca mammata*, and *Xylaria hypoxylon*), two *Leotiomycetes* (*Botrytis cinerea* and *Sclerotinia sclerotiorum*), and one Dothideomycete (*Zymoseptoria tritici*). A protein-coding super-alignment was constructed by identifying 43 single-copy orthologs from the fungi_odb10 BUSCO dataset that were complete, present in a single copy in all 14 taxa, and sufficiently conserved to yield reliable alignments. Individual loci were aligned using MAFFT before being concatenated for downstream analyses. The resulting super-alignment of 35.8 kbp was analyzed in two partitions corresponding to codon positions 1+2 and 3, each modeled with an unlinked Hasegawa–Kishino–Yano (HKY) substitution model and an uncorrelated lognormal relaxed clock. The tree prior was set to a Yule speciation process with a birth-rate parameter. Two calibrations were applied: (1) the crown group of *Xylariales* at 140 Ma (95% confidence interval [CI]: 77–219 Ma) (Samarakoon et al. 2016); and (2) the split between *Sordariomycetes* and *Leotiomycetes* with a median of 245 Ma (95% CI: 193–299 Ma) (Prieto and Wedin 2013). The final BEAST input file (*.xml) was generated in BEAUTi and analyzed with three independent runs of 100 million generations each. Convergence, effective sample sizes, burn-in, and 95% highest posterior density intervals were evaluated in Tracer v. 1.7.2 (Rambaut et al. 2018). Run results were combined in LogCombiner v. 1.10 after discarding the first 35 million generations from each run as burn-in. The resulting maximum clade credibility tree was visualized in FigTree v. 1.4.4 (Rambaut 2012).

### Mating-type locus identification

To identify the mating-type locus in *B. barriae*, the protein sequences XP_062685913.1 (putative DNA lyase APN2) and XP_062685910.1 (putative cytoskeleton assembly control protein SLA2) from *Neurospora tetraspora* were used as queries in tBLASTn searches against the genome assembly. Predicted proteins encoded within the candidate mating-type region were functionally annotated using InterProScan (Jones et al. 2014) to confirm the identity of the APN2 and SLA2 homologs and to characterize the putative mating-type gene.

## Results

### Species recognition and phylogenetic placement

**Figure 1.**
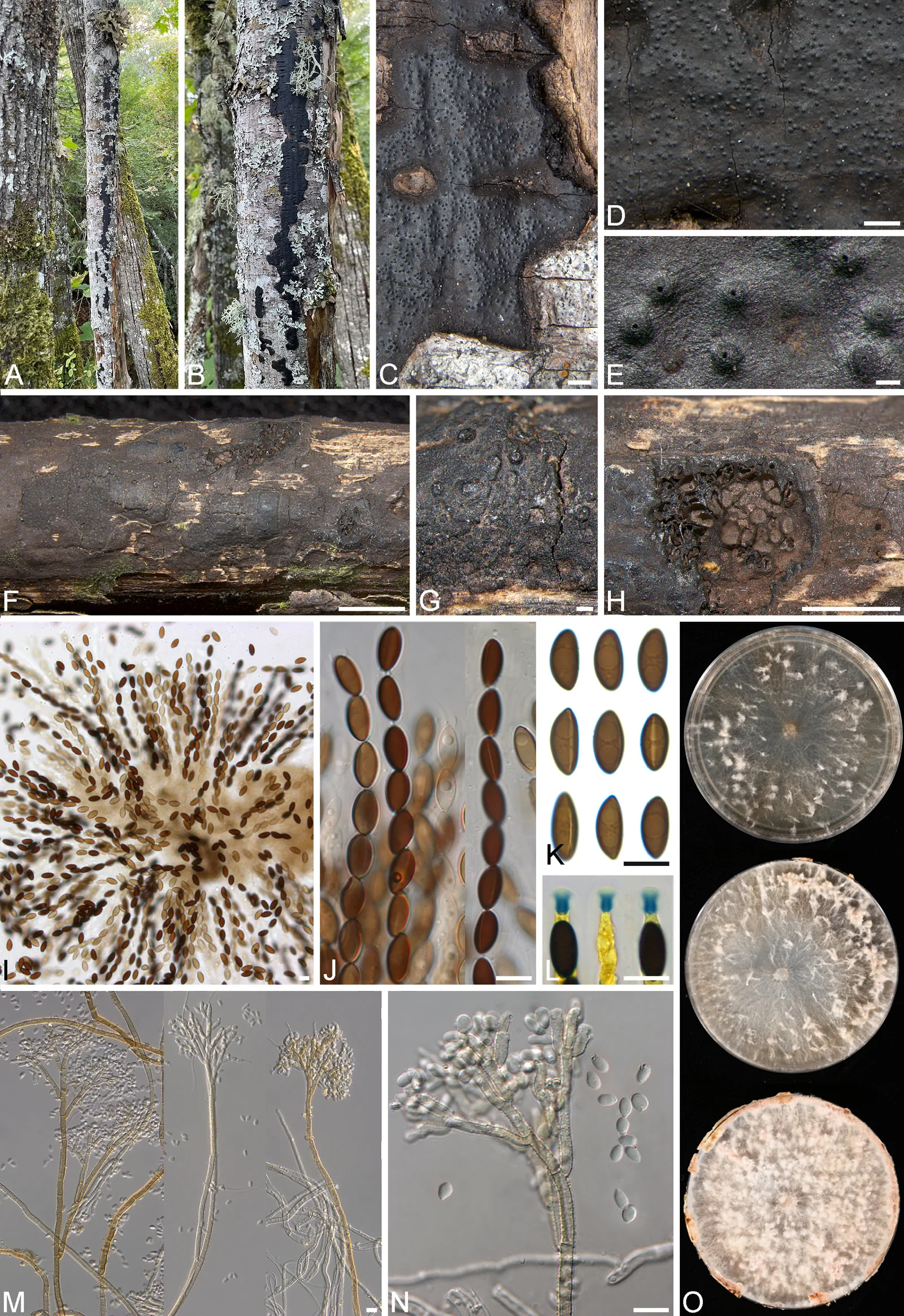
Morphology of *Biscogniauxia barriae*. **A–E**. DAVFP 30506 (holotype) stromata on dead standing bigleaf maple (*Acer macrophyllum*); **D–E**. Close-ups of perithecial ostioles on the stroma surface; **F–H**. WSP72952 stroma on a bigleaf maple branch; **I–J**. Asci and ascospores; **K**. Ascospores, some showing a distinct germ slit along the length of the spore; **L**. Ascus apical apparatuses stained blue in Lugol’s solution; **M–N**. Conidiophores and conidia from DAOMC 256939 grown on 2% malt extract agar; **O**. Three-week-old colonies of DAOMC 256939 on 2% malt extract agar (top), potato dextrose agar (middle), and oatmeal agar (bottom). Scale bars: 10 µm.

*Biscogniauxia* sp. nov. was recovered as an endophyte from surface-sterilized needles of Douglas-fir on southern Vancouver Island and from ascospores derived from stromata collected from dead bigleaf maple, red alder, and hawthorn in the same region (Fig. 1). Mono-ascospore cultures on 2% MEA were morphologically identical to the endophyte cultures, and both formed nodulisporium-like asexual morphs (Fig. 1M–O).

The primary phylogenetic dataset included 37 strains/specimens representing 28 taxa, including *Hypoxylon rubiginosum* (YMJ 24) and *Jackrogersella cohaerens* (YMJ 310), which were selected as outgroups. Single-gene alignments were generated for ITS, *BenA*, and *RPB2*, and taxa represented by all three loci were combined into a partitioned concatenated ITS–*BenA*–*RPB2* dataset. The concatenated alignment comprised 2,867 characters, of which 1,624 were constant, 266 were variable and parsimony-uninformative, and 977 were parsimony-informative. The final ML log-likelihood was −23,467.265. Alignment statistics and final ML optimization likelihoods for the individual gene trees are provided in Suppl. material 1.

**Figure 2.**
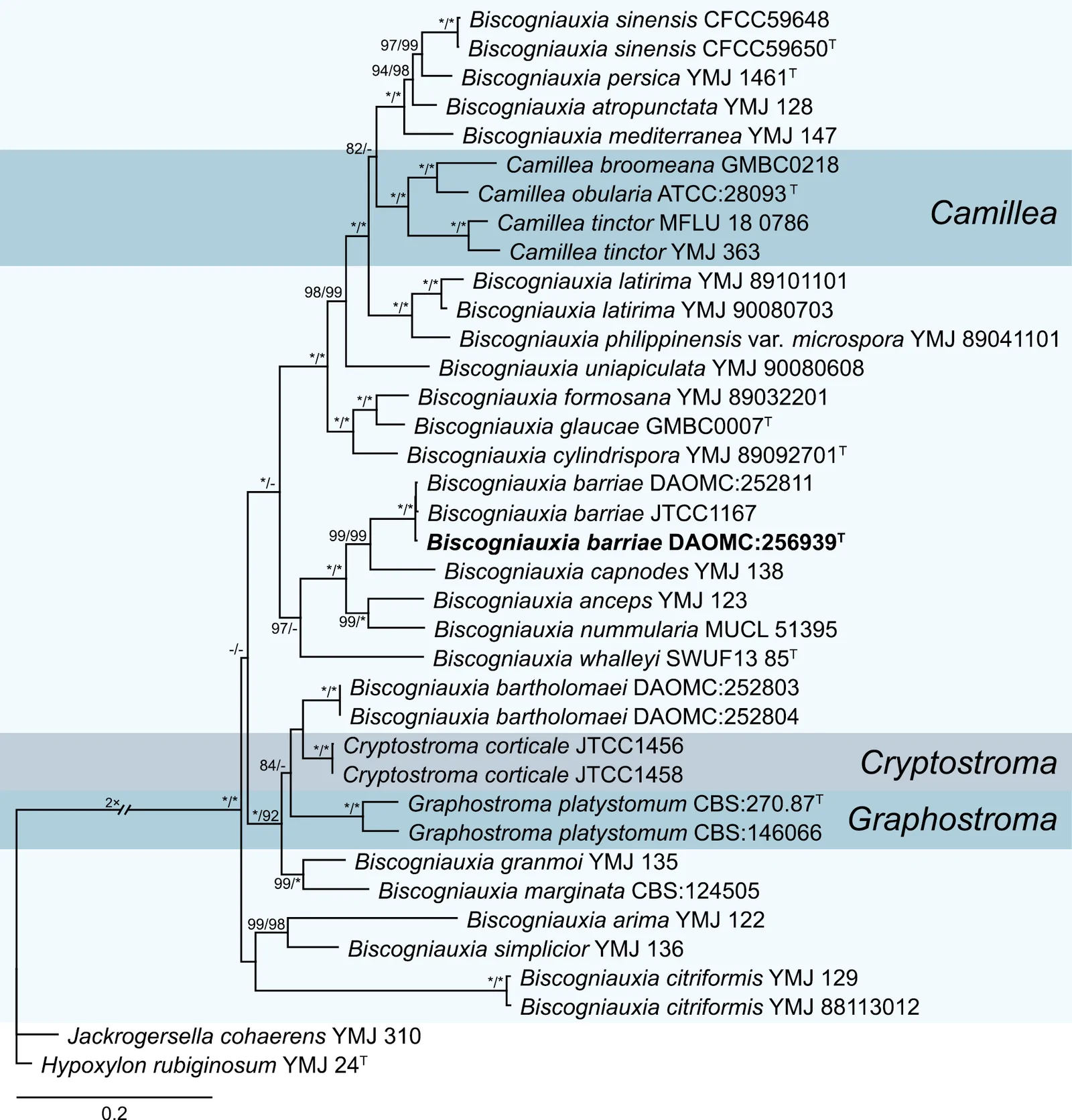
Maximum likelihood phylogenetic tree of *Graphostromataceae* based on a concatenated dataset consisting of ITS, *BenA*, and *RPB2*. The ex-type strain (DAOMC 256939) of *Biscogniauxia barriae* sp. nov. is denoted in bold. Binomial epithets are followed by strain or specimen ID, and sequences derived from type specimens are denoted with a superscript ‘^T^.’ Branch support values ≥80% SH-aLRT and ≥95% UFBoot are indicated at nodes, with asterisks denoting 100% support. *Hypoxylon rubiginosum* (YMJ 24) and *Jackrogersella cohaerens* (YMJ 310) (*Hypoxylaceae*, *Xylariales*) were selected as outgroup taxa. The scale bar represents substitutions per site.

In the concatenated analysis, strains of *Biscogniauxia* sp. nov. formed a distinct, well-supported clade separate from currently described species (Fig. 2). This clade was resolved as sister to *B. capnodes* within a strongly supported lineage that also included *B. anceps* and *B. nummularia*, with *B. whalleyi* placed basal to this group. ITS, *BenA*, and *RPB2* phylogenies were broadly congruent, with no strongly supported conflict affecting the placement of *Biscogniauxia* sp. nov. (Suppl. materials 2–4). A supplementary expanded concatenated analysis including 91 *Graphostromataceae* strains/specimens, with missing loci treated as missing data, recovered the same general placement while increasing representation of taxa and specimens not included in the complete three-locus dataset (Suppl. material 5). In this phylogeny, *B. barriae* was placed within a strongly supported clade with *B. capnodes*, *B. dicranopteridis*, *B. magna*, *B. mangiferae*, *B. papillata*, *B. petrensis*, and *B. xishuangbannaensis*, which was in turn sister to a clade containing *B. anceps* and *B. nummularia*, with *B. whalleyi* as the basal taxon.

### Genome sequencing and *de novo* assembly

**Figure 3.**
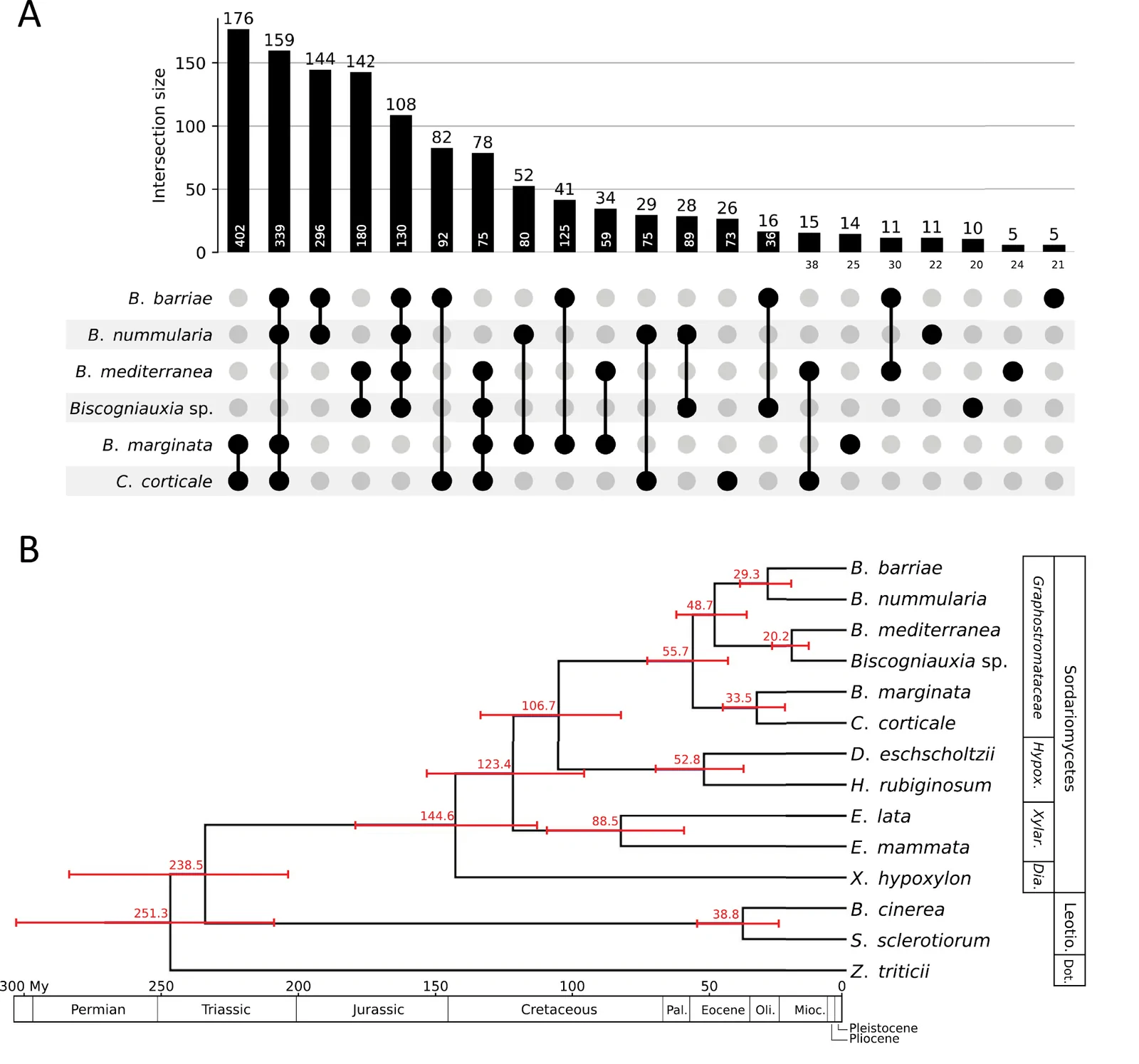
Divergence estimates and orthogroup sharing among *Biscogniauxia barriae* sp. nov. (DAOMC 256937) and related *Graphostromataceae* taxa. **A**. Number of orthogroups shared among *Biscogniauxia* spp. and *Cryptostroma corticale*, as inferred using OrthoFinder. Numbers of shared genes are indicated in white within bars or in black below bars; **B**. Divergence time estimates inferred from molecular clock analyses based on 43 nuclear protein-coding genes. Values at internal nodes indicate divergence times in millions of years (My), with red bars representing 95% highest posterior density intervals.

A consensus *de novo* assembly of 503 contigs totaling 42.7 Mb was generated, with a mean coverage of 276× and an N50 of 0.37 Mbp. Genome completeness analysis with the BUSCO fungi_odb10 dataset of 1,122 genes showed a completeness of 99.91%, which was higher than or comparable to assemblies reported for other species within *Biscogniauxia* (99.73–100%; Table 2). Using protein-coding gene models from three *Biscogniauxia* species, *ab initio* annotation of *B. barriae* predicted 10,986 protein-coding genes, with a BUSCO completeness of 99.91% (Table 2). This gene count is consistent with those reported for other *Biscogniauxia* species (10,869–11,671; Table 2), suggesting that the annotation of *B. barriae* did not result in substantial over- or underprediction. Clustering analysis performed with OrthoFinder indicated that 8,672 of these protein-coding genes, grouped into 7,895 orthogroups, were shared with five other *Graphostromataceae* species. In contrast, only five orthogroups comprising 21 protein-coding genes appeared to be unique to *B. barriae* (Fig. 3A). This number of species-unique protein-coding genes is in the same order as values observed for other *Biscogniauxia* species (5–14 orthogroups, representing 20–30 protein-coding genes per species). In addition, *B. barriae* shares 144 orthogroups (339 protein-coding genes) with its closest genome-sampled relative, *B. nummularia*, and 82 orthogroups (92 protein-coding genes) with the tree pathogen *C. corticale* (Fig. 3A). These patterns are consistent with their relative phylogenetic distances and estimated divergence times of 29.3 My (95% CI: 20.2–38.7 My) and ~55.7 My (43.4–72.5 My), respectively (Fig. 3B).

**Table 2.** Comparison of the general features of the genome sequence of *Biscogniauxia barriae* sp. nov. with other genomes of *Biscogniauxia* species. GenBank assembly accessions or JGI genome resource identifiers and associated references are provided where available. BUSCO categories: S, complete and single-copy; D, complete and duplicated; F, fragmented; M, missing.

	***B. barriae* sp. nov**.	***B. marginata***	***B. mediterranea***	***B. nummularia***	***Biscogniauxia* sp**.
Genome assembly/resource	JBZEML000000000	GCA_022695795.1	GCA_018398605.1	BnCUCC2015	GCA_022544895.1
Long reads (SRA)	SRR36946934	—	SRR14425514	SRR6448048-50	SRR10914828, SRR10914829
Short reads (SRA)	SRR36946933	SRR10842405	SRR14425513	—	—
Assembly size (Mbp)	42.7	45.6	43.7	41.2	41.7
Number of contigs	503	265	18	57	143
Coverage	275.9×	153.7×	60.0×	114.7×	317.6×
N50 (Mbp)	0.37	0.32	4.9	1.2	0.55
L50	30	41	5	11	24
Length of longest contig (Mbp)	1.76	1.68	5.87	3.71	2.89
No. of scaffolds > 1.0 Mbp	5	2	11	17	4
No. of scaffolds > 0.1 Mbp	118	127	14	46	96
GC content (%)	48.8	45.6	47.0	49.4	49.0
Masked nucleotides (%)	8.8	12.0	20.0	5.0	0.17
% BUSCO (S, D, F, M)	99.64, 0.0, 0.27, 0.09	99.64, 0.09, 0.27, 0.0	99.82, 0.0, 0.18, 0.0	99.38, 0.09, 0.27, 0.27	99.29, 0.36, 0.36, 0.0
Protein-coding genes	10,986	11,381	11,077	11,671	10,869
% BUSCO protein-coding genes (S, D, F, M)	96.7, 2.94, 0.27, 0.09	95.9, 3.21, 0.62, 0.27	96.26, 2.94, 0.53, 0.27	95.1, 3.39, 0.8, 0.71	95.9, 3.21, 0.53, 0.36
Genome reference	This study	Franco et al. 2022	Beccaccioli et al. 2021	Franco et al. 2022	Franco et al. 2022

tBLASTn searches using the APN2 and SLA2 proteins from *N. tetraspora* each produced a single highly significant hit located at the end of scaffold DF10_88 (APN2: 64% protein identity, *P* = 0.0; SLA2: 92%, *P* = 0.0). The recovery of a single APN2 and SLA2 homolog, both located on the same scaffold, is consistent with the presence of a single mating-type idiomorph in the assembled genome. InterProScan analysis identified a characteristic HMG-box DNA-binding domain in a hypothetical protein located between these flanking genes, supporting its annotation as the MAT1-2-1 mating-type gene.

### Taxonomy

#### Biscogniauxia barriae

Taxon classificationFungiXylarialesGraphostromataceae

J.B. Tanney and Y.M. Ju sp. nov.

9305B818-8B57-59F8-8435-BFFBEBEB7B24

864445

Figs 1, 2

##### Etymology.

Named in honor of Canadian mycologist Margaret Elizabeth Barr-Bigelow for her contributions to pyrenomycetes and in recognition of her initial collection of *B. barriae* (WSP72952).

##### Holotype.

Canada • British Columbia, Vancouver Island, Metchosin, 48°22'32.8"N, 123°33'37.4"W, 100 m elev., on bark of dead standing *Acer macrophyllum*, 24 February 2024, J.B. Tanney & B. Weir, DAVFP 30506 (preserved in a metabolically inactive state as dried specimen), living culture (ex-type) DAOMC 256939, GenBank numbers: PZ618325 (ITS), PZ618324 (LSU), PZ617968 (*BenA*), and PZ617971 (*RPB2*).

##### Description.

**Sexual morphology. *Stromata*** applanate to pulvinate, 4–170 mm long × 3–120 mm wide × 1–2 mm thick, with rounded to steep-sided margins, clearly demarcated from the substrate and sometimes slightly detached at the edge, frequently confluent; outer dehiscing layer not observed; mature surface dull to somewhat glossy, greyish brown to blackish grey or black; carbonaceous beneath the surface and between perithecia, with carbonaceous tissue encasing individual ostioles; tissue beneath perithecia 0.1–0.3 mm thick, carbonaceous; underlying bark tissue blackened, often conspicuously so between bark laminae. ***Perithecia*** short-cylindrical to obovoid or flask-shaped, base flattened, frequently laterally compressed, 0.6–0.8(–0.9) mm × 0.2–0.4 mm, opening centrally through individual ostioles, two adjacent perithecia rarely sharing a common ostiole. ***Ostioles*** concolorous with surrounding stroma, (20–)23–30(–40) μm diam, evenly distributed or in small clusters, coarsely papillate, (100–)138–182(–237) μm at base, up to 10 ostioles per 10 mm^2^. ***Asci*** cylindrical, 8-spored, ascospores arranged obliquely uniseriate, short-stipitate, (95–)100–120(–125) × (8–)9–11(–12) μm, pars sporifera (72–)75–94(–101) μm. ***Apical apparatus*** short-cylindrical to cuneiform or goblet-shaped, 2.5–6 × 3–6 μm (x̄ = 4.5 × 4.8 μm; *n* = 15), bluing in Lugol’s solution and Melzer’s reagent. ***Paraphyses*** sparse, hyphal, unbranched, thin-walled, septate, 4–5(–6) μm wide at base, tapering to 1 μm. ***Ascospores*** one-celled, broadly ellipsoid to somewhat ellipsoid-equilateral, (12.5–)14–15.5(–17) × (6–)6.5–7.5(–8.5) μm (x̄ = 14.6 × 6.9 μm; *n* = 153), *Q* = (1.7–)2–2.3(–2.7) (x̄ = 2.1), dark brown to blackish brown, smooth-walled, with a conspicuous, unilateral, straight germ slit running along the entire or almost the entire spore length on the most convex side, cellular appendage not observed.

##### Asexual morphology.

On 2% MEA: ***conidiophores*** nodulisporium-like, arising in loose tufts from aerial mycelia, hyaline to pale brown; stipes septate, upright, straight, 100–290 × 4–6 μm, hyaline to brown with maturity, smooth to roughened, branched. ***Conidiogenous cells*** cylindrical to clavate, hyaline to light brown, smooth to finely roughened, (6–)8–12.5(–20) × (2–)3–4(–4.5) μm (*n* = 103), bearing poroid conidial secession scars especially around the apical region. ***Conidia*** produced holoblastically in sympodial sequence, hyaline, smooth, obovoid to clavate, (4.5–)5.5–7(–9) × 3–4 μm, apiculate to flattened at base.

##### Culture characters.

Colonies on MEA at 20 °C in the dark reaching 74 mm diam or completely overgrowing 10-cm Petri dish in 7 d; after 7 d colonies flat, aerial mycelia white to faintly salmon, floccose, sometimes bearing honey to pink exudate droplets; white with hints of orange-pink, reverse white with occasional brown sectors. Colonies on PDA at 20 °C in the dark completely overgrowing 10-cm Petri dish in 7 d; after 7 d colonies flat, densely floccose to cottony, bearing honey to pink-red exudate droplets; white with reddish hues, reverse pink to ruby red from soluble pigments with occasional brown sectors. Colonies on OA at 20 °C in the dark completely overgrowing 10-cm Petri dish in 7 d; after 7 d colonies flat, aerial mycelia white to faintly salmon, floccose, sometimes bearing honey to pink exudate droplets; white with hints of orange-pink, reverse white with faint honey to brown hues.

##### Additional specimens examined.

Canada • British Columbia, Vancouver Island, Sidney, 48°38'29.0"N, 123°27'12.3"W, 32 m elev., dead young branches of *Acer macrophyllum*, 20 December 1997, M.E. Barr, WSP72952 • British Columbia, Vancouver Island, Metchosin, 48°22'03.2"N, 123°34'22.8"W, 120 m elev., on dead standing *Alnus rubra*, 14 April 2024, J.B. Tanney & B. Tanney, DAVFP 30507 (preserved in a metabolically inactive state as dried specimen), JBT-1036 • British Columbia, Vancouver Island, Saanich, 48°27'40.5"N, 123°23'42.5"W, 30 m elev., on dead branches of standing *Crataegus* sp., 15 July 2025, J.B. Tanney, DAVFP 30508 (preserved in a metabolically inactive state as dried specimen), JBT-1048 • British Columbia, Vancouver Island, Saanich, 48°27'40.5"N, 123°23'42.5"W, 30 m elev., on dead branches of standing *Crataegus* sp., 11 June 2026, J.B. Tanney, DAVFP 30509 (preserved in a metabolically inactive state as dried specimen), JBT-1069 • British Columbia, Vancouver Island, Metchosin, Witty’s Lagoon Regional Park, 48°23'04.3"N, 123°30'24.5"W, 14 m elev., isolated as endophyte from surface-sterilized *Pseudotsuga menziesii* needle fragment incubated on 2% malt extract agar, 16 June 2019, J.B. Tanney, DAOMC 256931 • *Ibid*. DAOMC 252811 • *Ibid*. DAOMC 256937 • *Ibid*. JTCC-837 • *Ibid*. JTCC-421 • British Columbia, Vancouver Island, Mesachie Lake, Cowichan Lake Research Station, 48°49'24.9"N, 124°08'37.6"W, 448 m elev., isolated as endophyte from surface-sterilized *Pseudotsuga menziesii* needle fragment incubated on 2% malt extract agar, 8 June 2022, J.B. Tanney, C. D’Amours, A. Dicaire, & V. Waring, JTCC-1167.

##### Habitat and distribution.

Foliar endophyte of *Pseudotsuga menziesii* and stromata from dead *Acer macrophyllum*, *Alnus rubra*, and *Crataegus* sp. on Vancouver Island, Canada; also reported from *A. macrophyllum* in Washington, USA (J.M. Hulbert, pers. comm.).

##### Notes.

The ITS–*BenA*–*RPB2* phylogeny places *Biscogniauxia barriae* within a clade containing *B. anceps*, *B. capnodes*, and *B. nummularia*. Morphologically, *B. barriae* is distinguished from these and other related species by its absence of hyaline septate ascospores (*B. anceps*) and longer ascospores: (12.5–)14–15.5(–17) × (6–)6.5–7.5(–8.5) μm vs. 8.5–15 × 5–7.5 μm (*B. capnodes*), 10–13(–14) × (6.5–)7.5–8.5 μm (*B. nummularia*), 11.5–14 × 6.5–8.5 μm (*B. magna*), 8.8–11.7 × 5.3–6.5 μm (*B. mangiferae*), and 11–15 × 5.7–7 μm (*B. petrensis*) (Samarakoon et al. 2022; Hyde et al. 2020; Ju et al. 1998; Rogers et al. 1996). Stromata of *B. barriae* are variable in size, forming small patches on branches and smaller stems and becoming extensive on larger stems.

## Discussion

*Biscogniauxia barriae* was discovered through the convergence of endophyte bioprospecting in Douglas-fir and forest disease surveys of declining maples, revealing a species with a dual life history as a conifer foliar endophyte and hardwood-associated stromatal fungus. Following the discovery of *B. barriae* stromata on bigleaf maple, the third author recalled an earlier collection from a nearby locality in Sidney, British Columbia, made in 1997 by M.E. Barr (WSP72952). That collection had been intended for description but remained unpublished. Here, *B. barriae* is described in honor of Margaret Elizabeth Barr-Bigelow, based on morphological and phylogenetic evidence supporting its recognition as a distinct species.

The first route to recognizing *B. barriae* was through Douglas-fir endophyte research. *Biscogniauxia barriae* was repeatedly isolated as a foliar endophyte of asymptomatic Douglas-fir needles (Graham et al. 2025). Notably, culture-filtrate extracts of two *B. barriae* strains showed the strongest inhibition of the *in vitro* growth of *Nothophaeocryptopus gaeumannii*, the causal agent of Swiss needle cast of Douglas-fir. These results suggested that the strains represented a novel and potentially ecologically relevant member of the Douglas-fir needle mycobiome, prompting passive monitoring of sympatric hardwoods for corresponding stromata to aid in its description.

The second route to recognizing *B. barriae* was through maple disease surveillance. Ongoing work on *Cryptostroma corticale*, causal agent of an emerging disease of maple (*Acer*) trees in coastal British Columbia, Canada, stimulated interest in *Graphostromataceae* in general and led to surveys of bigleaf maple (*A. macrophyllum*) for *C. corticale* and related species (Tanney et al. 2024). Stromata of *Biscogniauxia barriae* were discovered on dead bigleaf maple trees and subsequently cultured to assess conspecificity with Douglas-fir endophyte strains and to evaluate relatedness to *C. corticale*. Because *B. barriae* and *C. corticale* occur sympatrically on maple in coastal British Columbia, they may be encountered during the same surveys of declining or recently dead trees. However, they represent distinct *Graphostromataceae* lineages and can be separated readily by morphology: *B. barriae* produces perithecial stromata with visible ostioles, which can be observed using a hand lens in the field (Fig. 1C–E), whereas sexual reproduction has not been reported in *C. corticale*, and its stromata therefore lack perithecia and ostioles. This distinction is also relevant diagnostically, as *B. barriae* showed cross-reactivity in an earlier nested PCR assay developed for *C. corticale*, although the improved assay described by Dicaire et al. (2025) avoids this cross-reactivity.

The description of *B. barriae* documents another case of an endophyte inhabiting conifer foliage and exhibiting sexual reproduction during a saprotrophic phase on alternate hosts or substrates (Tanney et al. 2016; Tanney and Seifert 2020). Similar life history patterns have been reported for *Xylaria ellisii*, which occurs as an endophyte on diverse host leaves (e.g., *Picea*, *Pinus*, *Vaccinium*, and *Vitis*) and as a saprotroph producing stromata on decaying hardwood trees (e.g., *Acer*, *Betula*, and *Fagus*) (Ibrahim et al. 2020). This species produced antifungal secondary metabolites, including griseofulvin, at biologically effective concentrations within *Pinus strobus* needles. Although the ecological role of *B. barriae* in Douglas-fir needles remains unresolved, strong inhibition of the *in vitro* growth of *Nothophaeocryptopus gaeumannii* by culture-filtrate extracts of *B. barriae* suggests potential ecological relevance within the Douglas-fir needle mycobiome and the Swiss needle cast pathosystem. Future work should identify and characterize the bioactive natural products responsible for this activity.

The nature of host interactions in *B. barriae* remains unresolved. The species was isolated from asymptomatic Douglas-fir needles, and preliminary inoculation trials on Douglas-fir germinants and seedlings did not indicate pathogenicity (unpublished data). A recently described species, *B. sinensis*, was isolated from *Pinus thunbergii* needles showing blight symptoms; however, pathogenicity tests do not appear to have been conducted, and therefore, it cannot be considered a causal agent without additional evidence (Qiao et al. 2024).

The ITS–*BenA*–*RPB2* phylogeny places *B. barriae* within a strongly supported clade that is interpreted as *Biscogniauxia* s.s. based on its inclusion of the ex-epitype strain (MUCL 51395) of the type species *B. nummularia* (Fig. 2). As observed by previous authors, the current broad circumscription of *Biscogniauxia* is polyphyletic, with *Camillea*, *Cryptostroma*, and *Graphostroma* nested among lineages currently assigned to *Biscogniauxia* (Fig. 2). The *Graphostromataceae* genus *Obolarina* was distinguished from *Biscogniauxia* by its larger ascospores with spiral germ slits and lack of a distinct ascal apical ring (Ju et al. 1998). The genus included two species, the type species *O. dryophila* and *O. persica*, both of which were transferred to *Biscogniauxia* by Bashiri et al. (2022). However, this taxonomic decision has not been fully adopted by subsequent authors (e.g., Liu et al. 2026; Wongkanoun et al. 2026). Overall, further work is needed to establish generic limits within *Graphostromataceae*, particularly the relationships among *Biscogniauxia*, *Camillea*, and *Cryptostroma*.

The genome assembly of *B. barriae* is consistent with its placement within *Biscogniauxia* and provides a genomic baseline for future studies of its ecology and secondary metabolism. Genome size, GC content, BUSCO completeness, and predicted gene number were broadly comparable to available genomes of other *Biscogniauxia* species, indicating that *B. barriae* falls within the range of conserved genomic architecture observed across the genus. However, while completeness was high, the draft assembly remains moderately fragmented (N50 0.37 Mbp), which may limit resolution of large-scale genomic features such as complete secondary metabolite gene clusters. Orthogroup comparisons likewise suggested extensive conservation of protein-coding gene content, with relatively few species-unique orthogroups detected in *B. barriae*, suggesting that ecological or phenotypic differences among species may arise from regulatory or functional divergence rather than major differences in gene repertoire. The identification of a single MAT1-2 idiomorph is consistent with a heterothallic mating system, with potential implications for population structure and reproduction. Finally, the estimated divergence of *B. barriae* from *B. nummularia* at approximately 29 Ma further supports its recognition as an evolutionarily distinct lineage within the genus. In contrast, the deeper divergence between *B. barriae* and *C. corticale* is consistent with the phylogenetic analyses and reinforces that these sympatric maple-associated *Graphostromataceae* represent distinct evolutionary lineages. Together, these results provide a framework for future comparative analyses aimed at identifying genes or metabolite pathways associated with host association, latent pathogenicity, saprotrophy, and antifungal activity.

*Biscogniauxia barriae* forms extensive stromata on recently dead bigleaf maple and has also been collected from red alder and hawthorn. Although its role in hardwood decline remains unresolved, the occurrence of stromata on recently dead hosts, the known latent-pathogenic behavior of some *Biscogniauxia* species, and the emergence of heat- and drought-associated *Graphostromataceae* diseases in the Pacific Northwest warrant pathogenicity assays on bigleaf maple under stress conditions. Overall, *B. barriae* appears to occupy different ecological roles across hosts: an asymptomatically occurring foliar endophyte in Douglas-fir, where its interaction with the host remains unknown, and a saprotroph on dead hardwoods or a possible opportunistic latent pathogen in stressed hardwoods.

## Supplementary Material

XML Treatment for Biscogniauxia barriae

## Additional information

### Artificial Intelligence (AI) use

The authors accept full responsibility for the content of the manuscript, including the disclosure of any use of AI.

No AI tools were used in the preparation of this manuscript.

### Author ORCIDs

J.B. Tanney https://orcid.org/0000-0002-6151-9346

N. Feau https://orcid.org/0000-0001-5925-9867

Y.-M. Ju https://orcid.org/0000-0002-8202-6145

### Data availability

All of the data that support the findings of this study are available in the main text or Supplementary Information.

## Supplementary materials

Supplementary material 1 Alignment statistics, best-fit substitution models, and final ML optimization likelihoods for the single-locus and concatenated datasets Data type xlsx https://binary.pensoft.net/file/1767495This dataset is made available under the Open Database License (http://opendatacommons.org/licenses/odbl/1.0/). The Open Database License (ODbL) is a license agreement intended to allow users to freely share, modify, and use this Dataset while maintaining this same freedom for others, provided that the original source and author(s) are credited. Joey B. Tanney, Nicolas Feau, Yu-Ming Ju

Supplementary material 2 ML phylogenetic tree of *Graphostromataceae* based on ITS sequences Data type svg Explanation note The ex-type strain (DAOMC 256939) of *Biscogniauxia barriae* sp. nov. is denoted in bold. Binomial epithets are followed by strain or specimen ID, and sequences derived from type specimens are denoted with a superscript ‘^T^.’ Branch support values ≥80% SH-aLRT and ≥95% UFBoot are indicated at nodes, with asterisks denoting 100% support. *Hypoxylon rubiginosum* (YMJ 24) and *Jackrogersella cohaerens* (YMJ 310) were selected as outgroup taxa. https://binary.pensoft.net/file/1767496This dataset is made available under the Open Database License (http://opendatacommons.org/licenses/odbl/1.0/). The Open Database License (ODbL) is a license agreement intended to allow users to freely share, modify, and use this Dataset while maintaining this same freedom for others, provided that the original source and author(s) are credited. Joey B. Tanney, Nicolas Feau, Yu-Ming Ju

Supplementary material 3 ML phylogenetic tree of *Graphostromataceae* based on *BenA* sequences Data type svg Explanation note The ex-type strain (DAOMC 256939) of *Biscogniauxia barriae* sp. nov. is denoted in bold. Binomial epithets are followed by strain or specimen ID, and sequences derived from type specimens are denoted with a superscript ‘^T^.’ Branch support values ≥80% SH-aLRT and ≥95% UFBoot are indicated at nodes, with asterisks denoting 100% support. *Hypoxylon rubiginosum* (YMJ 24) and *Jackrogersella cohaerens* (YMJ 310) were selected as outgroup taxa. https://binary.pensoft.net/file/1767497This dataset is made available under the Open Database License (http://opendatacommons.org/licenses/odbl/1.0/). The Open Database License (ODbL) is a license agreement intended to allow users to freely share, modify, and use this Dataset while maintaining this same freedom for others, provided that the original source and author(s) are credited. Joey B. Tanney, Nicolas Feau, Yu-Ming Ju

Supplementary material 4 ML phylogenetic tree of *Graphostromataceae* based on *RPB2* sequences Data type svg Explanation note The ex-type strain (DAOMC 256939) of *Biscogniauxia barriae* sp. nov. is denoted in bold. Binomial epithets are followed by strain or specimen ID, and sequences derived from type specimens are denoted with a superscript ‘^T^.’ Branch support values ≥80% SH-aLRT and ≥95% UFBoot are indicated at nodes, with asterisks denoting 100% support. *Hypoxylon rubiginosum* (YMJ 24) and *Jackrogersella cohaerens* (YMJ 310) were selected as outgroup taxa. https://binary.pensoft.net/file/1767498This dataset is made available under the Open Database License (http://opendatacommons.org/licenses/odbl/1.0/). The Open Database License (ODbL) is a license agreement intended to allow users to freely share, modify, and use this Dataset while maintaining this same freedom for others, provided that the original source and author(s) are credited. Joey B. Tanney, Nicolas Feau, Yu-Ming Ju

Supplementary material 5 Expanded ML phylogenetic tree of *Graphostromataceae* based on a partitioned concatenated ITS–*BenA*–*RPB2* dataset Data type svg Explanation note Taxa represented by one, two, or all three loci were included, with unavailable gene regions treated as missing data. The ex-type strain (DAOMC 256939) of *Biscogniauxia barriae* sp. nov. is denoted in bold. Binomial epithets are followed by strain or specimen ID, and sequences derived from type specimens are denoted with a superscript ‘^T^.’ Branch support values ≥80% SH-aLRT and ≥95% UFBoot are indicated at nodes, with asterisks denoting 100% support. *Hypoxylon rubiginosum* (YMJ 24) and *Jackrogersella cohaerens* (YMJ 310) were selected as outgroup taxa. https://binary.pensoft.net/file/1767499This dataset is made available under the Open Database License (http://opendatacommons.org/licenses/odbl/1.0/). The Open Database License (ODbL) is a license agreement intended to allow users to freely share, modify, and use this Dataset while maintaining this same freedom for others, provided that the original source and author(s) are credited. Joey B. Tanney, Nicolas Feau, Yu-Ming Ju
